# The complete chloroplast genome sequence of medicinal plant *Alpinia chinensis* (Retz.) Rosc

**DOI:** 10.1080/23802359.2020.1817805

**Published:** 2020-09-11

**Authors:** Yu Mei, Shike Cai, Shiqiang Xu, Yan Gu, Fang Zhou, Jihua Wang

**Affiliations:** Guangdong Key Laboratory of Crops Genetic Improvement, Crops Research Institute, Guangdong Academy of Agricultural Sciences, Guangzhou, P. R. China

**Keywords:** *Alpinia chinensis*, chloroplast genome, medicinal plant

## Abstract

*Alpinia chinensis* (Retz.) Rosc is one of Chinese tradition herbal medicine and edible plant in China. In this report, we sequenced the complete chloroplast genome of *A. chinensis*. Through the assembly annotation of genome with high-throughput sequencing data, which help us to research the evolution. The length of chloroplast sequences was 163,590 bp with a large single-copy region (LSC) and a small single-copy region (SSC), also, two inverted repeat region A (IR), whose length was 88,951, 15,299, and 29,670 bp, respectively. A total of 138 genes were predicted in the complete chloroplast genome, with 36.4% GC content, including 93 protein-coding genes, 37 tRNA genes, and 8 rRNA genes. From the phylogenetic analysis, we could conclude that *A. chinensis* (Retz.) Rosc. was close to *Alpinia oxyphylla* in Zingiberaceae.

*Alpinia chinensis* belongs to Zingiberaceae family, widely distributed in southeast to southwest provinces of China, which is a famous national medicinal with multiple medicinal properties (Qi et al. 2020). *Alpinia chinensis* (Retz.) contain abundant chemical composition, of which most are medical resource such as volatile oils, in the roots, leaves, and seeds. The chloroplast (cp) is an important organelle in plant cells, which can produce essential energy through photosynthesis and has its own independent genetic material (Daniell et al. 2016). The cp genome provides valuable information for species identification and phylogenetic analysis (Shen et al. 2017). In recent years, researchers have reported the cp genome of medicinal plants. Whereas, the cp genome of *A. chinensis* has not been reported.

In this study, the complete cp genome of *A. chinensis* was reported. The leaves of *A. chinensis* were collected at the Fengwan town, Shaoguan city, Guangdong province, China (E113°51′ 26.9496″, N24°48′42.2712″). The samples were frozen by liquid nitrogen and stored at −80 °C refrigerator before extracting genomic DNA using plant genomic DNA kit (Omega, USA), then constructed DNA library with an average insert size 350-bp using VAHTS Universal DNA Library Prep Kit (Vazyme ND606-01, Nanjing, China), which were kept at Key Laboratory for Crops Genetic Improvement of Guangdong in Guangdong Academy of Agricultural Sciences (specimen code Hsj2020). Finally, the library was sequenced on the Novaseq platform (Illumina, San Diego, CA, USA). The sequence was assembled and annotated by GetOrganelle (Jin et al. 2019) and Geseq (Tillich et al. 2017), using the cp annotation of *Alpinia oxyphylla* (KY985237) as a reference. Then, submitted to GenBank with the accession number MT239402.

The length of the cp genome sequence was 163,590 bp, with 36.4% GC content, consisted of a large single-copy region (LSC) region (88,951 bp) and small single-copy region (SSC) region (15,299 bp), separated by a pair of inverted repeat region (IR) region (29,670 bp). A total of 138 genes were predicted in the cp genome of *A. chinensis*, including 93 protein-coding genes, 37 tRNA genes, and 8 rRNA genes. Through RaxML software, the complete chloroplast genomes of *A. chinensis* and other 18 species were constructed by the maximum likelihood method using the GTRGAMMA nucleotide substitution model (Stamatakis 2014). So in Figure 1, We could conclude that *A. chinensis* was close to *A. oxyphylla* in Zingiberaceae. These researches will provide valuable genomic information for the barcode and make an essential foundation for molecular breeding and genetic engineering of *A. chinensis* in the future.

**Figure 1.**
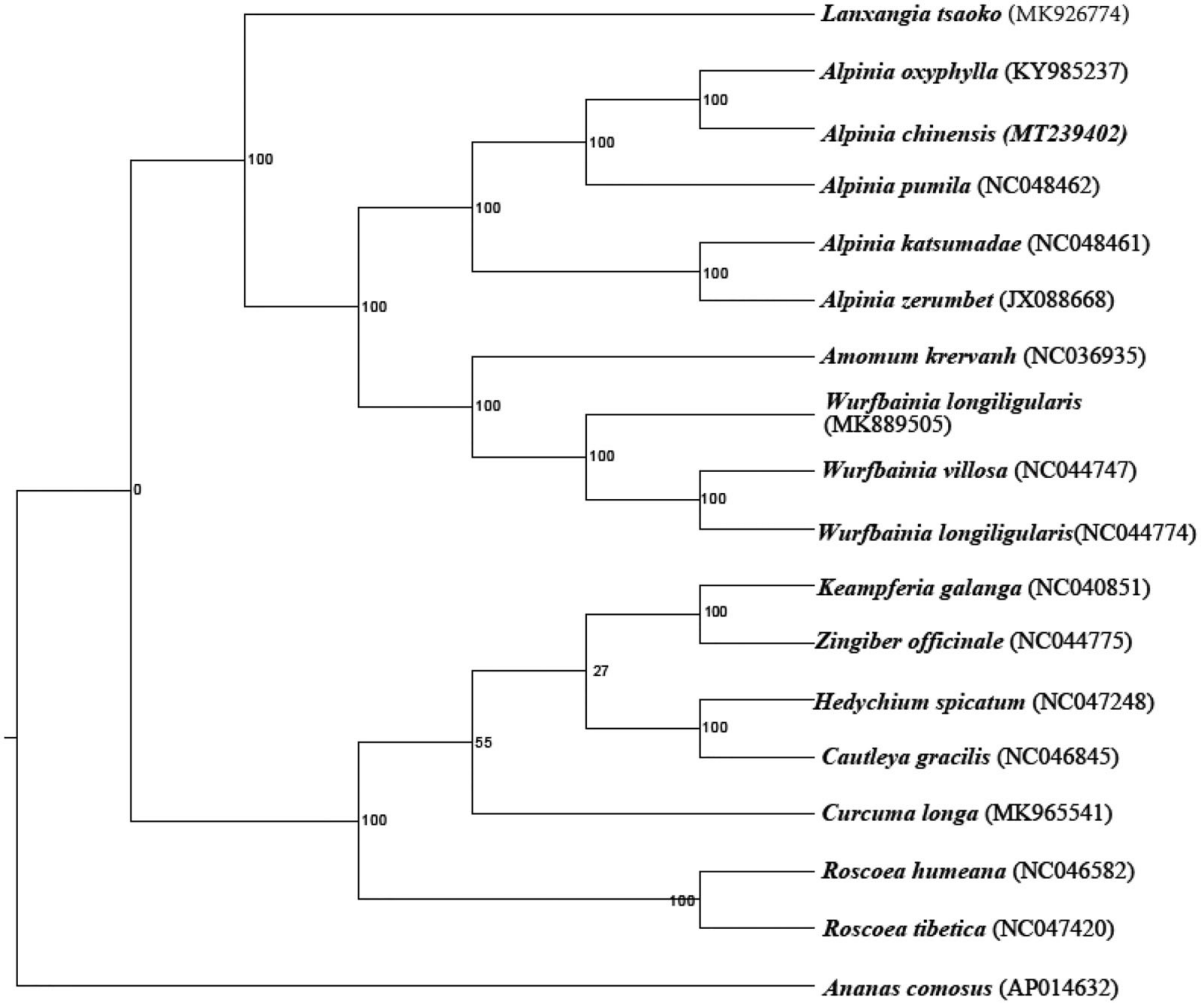
The phylogenetic tree of the *A. chinensis* and other species based on the 18 complete chloroplast genome sequences with 1000 bootstrap replicates.

## Data Availability

The data that support the findings of this study are openly available in [GenBank] at [https://www.ncbi.nlm.nih.gov/nuccore/MT239402.1], reference number [MT239402.1].
